# Diabetic myopathy: impact of diabetes mellitus on skeletal muscle progenitor cells

**DOI:** 10.3389/fphys.2013.00379

**Published:** 2013-12-20

**Authors:** Donna M. D'Souza, Dhuha Al-Sajee, Thomas J. Hawke

**Affiliations:** Department of Pathology and Molecular Medicine, McMaster UniversityHamilton, ON, Canada

**Keywords:** diabetes mellitus, muscle satellite cells, PICs, skeletal muscle, muscle regeneration, Type 1 diabetes mellitus, Type 2 diabetes mellitus

## Abstract

Diabetes mellitus is defined as a group of metabolic diseases that are associated with the presence of a hyperglycemic state due to impairments in insulin release and/or function. While the development of each form of diabetes (Type 1 or Type 2) drastically differs, resultant pathologies often overlap. In each diabetic condition, a failure to maintain healthy muscle is often observed, and is termed diabetic myopathy. This significant, but often overlooked, complication is believed to contribute to the progression of additional diabetic complications due to the vital importance of skeletal muscle for our physical and metabolic well-being. While studies have investigated the link between changes to skeletal muscle metabolic health following diabetes mellitus onset (particularly Type 2 diabetes mellitus), few have examined the negative impact of diabetes mellitus on the growth and reparative capacities of skeletal muscle that often coincides with disease development. Importantly, evidence is accumulating that the muscle progenitor cell population (particularly the muscle satellite cell population) is also negatively affected by the diabetic environment, and as such, likely contributes to the declining skeletal muscle health observed in diabetes mellitus. In this review, we summarize the current knowledge surrounding the influence of diabetes mellitus on skeletal muscle growth and repair, with a particular emphasis on the impact of diabetes mellitus on skeletal muscle progenitor cell populations.

## Skeletal muscle and muscle progenitor cells

Skeletal muscle is capable of adapting to numerous stimuli, with these responses manifested through changes in muscle size, fiber-type distribution, and/or metabolism. A critical component in skeletal muscle maintenance and plasticity is the presence of muscle progenitor cells. A complex network of intrinsic and extrinsic factors mediate changes to these progenitor cells, with such factors influenced by, and influential to, skeletal muscle health. Diseases that negatively impact muscle health, such as diabetes mellitus, may do so by negatively affecting progenitor cell quantity and/or functionality. As such, these cells (or a sub-population therein) function as a primary therapeutic target to attenuate deficits in muscle health with disease progression. While the most well defined of these progenitor cells is the satellite cell (SC; Hawke and Garry, 2001; Zammit and Relaix, 2012), evidence of a number of non-satellite cell progenitor populations contributing to the maintenance of skeletal muscle in health and disease has emerged in recent years (Pannérec et al., 2012). Throughout this brief review we will use the terms “satellite cells” or “SCs” to define this heterogeneous progenitor cell population, acknowledging that as our understanding of these other unique cell populations becomes clearer, we may revisit roles once allocated specifically to the muscle satellite cells.

## Pathophysiology of type 1 and 2 diabetes mellitus

The onset of Type 1 diabetes mellitus (T1DM) often occurs in childhood or adolescence and is characterized by the immune-mediated destruction of pancreatic β-cells leading to insulin deficiency. While T1DM accounts for only ~10% of diabetic cases, its prevalence over the past 30 years has increased worldwide (Onkamo et al., 1999; Gale, 2002). Adolescent muscles subjected to atrophic stimuli are more likely to endure irreversible changes (Darr and Schultz, 1989; Mozdziak et al., 2000), thus the presentation of T1DM during this critical growth period can detrimentally impact long-term muscle health. In contrast, Type 2 Diabetes Mellitus (T2DM) accounts for ~90% of diabetes mellitus cases (Masso-Gonzalez et al., 2009), and is expected to affect almost 8% of the worldwide population by 2030 (Shaw et al., 2010). Adverse health behaviors, particularly sedentary lifestyles and increased adiposity, have lead to a high incidence of insulin resistance and impaired fasting glucose (American Diabetes Association, 2006). Without therapeutic intervention, the insulin-resistant state often precipitates to pancreatic β-cell death and progression to insulin-dependent T2DM.

While the etiology of T1DM and T2DM are distinct, the end result is number of common co-morbidities including nephropathy, neuropathy, and cardiovascular disease. Diabetic myopathy, characterized by reduced physical capacity, strength, and muscle mass (Andersen et al., 1996, 1997, 2004, 2005), is a relatively understudied complication of diabetes mellitus, but is believed to directly influence the rate of co-morbidity development. This is based on the fact that skeletal muscle functions as the largest site for glucose uptake (DeFronzo et al., 1981), and therefore changes to skeletal muscle health can impact whole-body glucose homeostasis. A vital component to the maintenance of skeletal muscle is its SC population. As such, changes to SC functionality with diabetes mellitus would impact skeletal muscle health. Here we review the current state of knowledge on the relationship between skeletal muscle health and diabetes mellitus, with a particular focus on the fate and function of skeletal muscle progenitor cell populations.

## Skeletal muscle in diabetes mellitus

### T1DM

Muscle growth and development is significantly impaired in T1DM, resulting in reduced muscle mass and myofiber size, poor metabolic control, and a switch to a glycolytic phenotype (Andersen et al., 1997, 2004; Crowther et al., 2003; Fritzsche et al., 2008; Krause et al., 2009, 2013). While initial studies in human T1DM reported no difference in capillary density (Leinonen et al., 1982), investigations in T1DM mice illustrate that the disease is associated with a decline in skeletal muscle capillarization and angiogenesis (Kivelä et al., 2006; Krause et al., 2009). These alterations to muscle structure and metabolism often are associated with reductions in muscle function, as previously demonstrated (Huttunen et al., 1984; Poortmans et al., 1986; Almeida et al., 2008; Gordon et al., 2010). In addition to growth and function, the capacity for repair from damage is also adversely affected by T1DM, as indicated by studies of muscle regeneration using chemical and genetic models of T1DM (Gulati and Swamy, 1991; Talesara and Vashishta, 2000; Vignaud et al., 2007; Krause et al., 2011, 2013). Collectively, these studies highlight the negative impact T1DM is having on skeletal muscle and its potential for growth, maintenance, and repair.

### Satellite cells and T1DM

Satellite cells from streptozotocin (STZ)-treated diabetic mice fail to activate properly, resulting in failed regeneration following chemically induced muscle injury (Jeong et al., 2013). This extreme catabolic state has previously been shown to promote the fusion of SCs to adjacent muscle fibers in T1DM mice, as this is thought to promote the release of factors that function to sustain muscle integrity in this less than favorable metabolic condition (Brannon et al., 1989). Furthermore, Aragno et al. (2004) reported reduced myogenic regulatory factor expression and impaired differentiation in T1DM-derived myoblasts. Attenuated muscle repair has also been observed in T1DM mice (Krause et al., 2011, 2013). The impaired regeneration with diabetes was attributed to an elevation in plasma PAI-1 resulting from attenuated extracellular matrix (ECM) turnover. The delay in ECM turnover inhibited macrophage and SC migration into the damaged/necrotic regions of injured muscle. Interestingly, despite systemic increases in PAI-1, the impaired regeneration occurred in a muscle-specific pattern (Krause et al., 2013), indicating that muscles are intrinsically resistant to the T1DM environment. It is becoming increasingly clear that alterations to muscle protein turnover cannot, by itself, account for diabetic myopathy. Although studies investigating SCs in T1DM remains limited, evidence indicates that functionality is affected. Clinically, it is important to appreciate that T1DM-onset almost always occurs during childhood/adolescence, a period of extensive muscle growth. Thus, understanding alterations to the SC population in T1DM is essential for the development of therapeutic strategies to maximize muscle health during this vulnerable time.

### T2DM

Similar to observations in T1DM, skeletal muscle of T2DM subjects exhibit increased glycolytic fiber number (Mårin et al., 1994; Nyholm et al., 1997), muscle atrophy (Huang et al., 2010), and decreases in capillary density (Prior et al., 2009). Perturbations to muscle metabolism in T2DM are common, resulting in decreased intermyofibrillar mitochondrial content and abnormal lipid deposition (Nielsen et al., 2010; Chomentowski et al., 2011). As a consequence of these unfavorable changes, the muscle becomes “metabolically inflexible” as it cannot easily switch between fat and carbohydrate oxidation in response to insulin (Kelley and Mandarino, 2000). Functional impairments are also evident, as demonstrated by a decline in muscle strength (Andersen et al., 2004; Park et al., 2006), a finding strongly correlated with intramuscular fat storage (Hilton et al., 2008). Studies of muscle regeneration in insulin-resistance/T2DM animal models (Nguyen et al., 2011) further identify attenuated skeletal muscle plasticity, with deleterious changes to SC function theorized as a central mechanism underlying the observed outcomes.

As discussed, diabetes mellitus impinges on skeletal muscle health. Studies have noted that the diabetic environment enhances protein degradation (Price et al., 1996; Lecker et al., 1999; Mitch et al., 1999; Mastrocola et al., 2008). While these studies are well conducted, it was not within their scope to investigate all components required for skeletal muscle growth and maintenance. Indeed, SCs are indispensable for such events (Zammit and Relaix, 2012), and thus a more complete understanding of the impact of the diabetic environment on SC function is needed.

### Satellite cells and T2DM

While studies directly assessing SC function with T2DM remain limited, a number of recent investigations have evaluated SC behavior with hyperglycemia and/or lipotoxicity. For instance, 3 weeks of a high fat feeding (HFF) affected SC content and functionality, with the latter classified as the quantity of regenerating fibers present following injury (Fitzpatrick et al., 2011). Hu et al. (2010) demonstrated reduced muscle regeneration after 8-months HFF that was attributed to a delay in myofiber maturation, rather than SC activation or proliferation. *In vitro* studies have also shown that SCs incubated in high glucose medium have an increased propensity to differentiate into adipocytes (Aguiari et al., 2008), suggesting that SC myogenic capacity may be impacted by uncontrolled diabetes. This is further substantiated through the use of genetic models of obesity and diabetes. The Obese Zucker Rat (OZR), a model for the metabolic syndrome, displays reduced SC proliferative capacity though quiescent SC percentages remain unchanged (Peterson et al., 2008); findings consistent with observed alterations to Akt signaling and myogenic regulatory factor expression (Peterson et al., 2008). Similar results were obtained in transgenic (*ob/ob*, *db/db*) models of T2DM. Specifically, impaired SC proliferation and activation were observed and were reflected in measurable impairments of muscle regeneration (Nguyen et al., 2011). A critical, but as of yet unanswered, question is the role of altered leptin signaling in mediating changes to SCs in these animal models. Interestingly, these authors found no difference in SC function or regenerative capacity in HFF mice (Nguyen et al., 2011).

In addition to altered myogenic potential, SCs derived from T2DM patients were found to retain a “diabetic phenotype” upon isolation and culturing. These T2DM-derived SCs displayed reduced lipid oxidation (Gaster et al., 2004), increased secretion of inflammatory markers leading to altered cell signaling (Green et al., 2011), impaired glucose transport (Gaster et al., 2002), and insulin-resistance (Scarda et al., 2010). These modifications were based on T2DM-induced epigenetic changes to muscle cell gene programming, modifying protein expression of factors essential to myogenesis, thereby permanently affecting muscle SCs (Broholm et al., 2012). Taken together, these findings suggest that the degree of T2DM disease severity (i.e., diet vs. genetic model) will differentially influence SC function. A more severe T2DM phenotype, as is found in genetic models of T2DM, results in impairments to the early stages of myogenesis (proliferation, activation), while the HFF models will alter the differentiation potential of the SCs. Finally, long-term exposure to T2DM may promote detrimental epigenetic changes to SCs that will inevitably affect their functionality, and ultimately, overall skeletal muscle health.

## Mechanisms for altered satellite cell function in diabetes mellitus

The literature is clear that the uncontrolled diabetic environment is unfavorable for skeletal muscle growth and regeneration (Vignaud et al., 2007; Peterson et al., 2008; Krause et al., 2011; Nguyen et al., 2011). However, the molecular processes governing changes to the SCs are far from elucidated.

**Figure 1:**
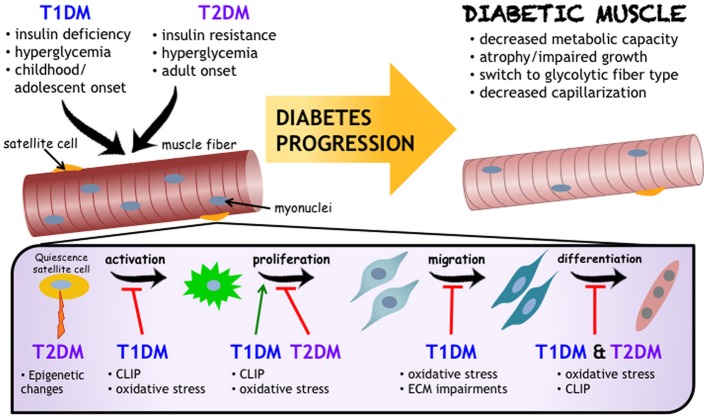
**Impact of Diabetes Mellitus on Skeletal Muscle Health.** While the etiology and progression for T1DM and T2DM development are distinct, both diseases negatively influence skeletal muscle (referred to as “Diabetic Muscle”) and their resident progenitor cell populations, including satellite cells. Satellite cells are critical to muscle health, and are affected by diabetes mellitus at varying stages of adult myogenesis. As outlined in this review, and schematized here, chronic low grade inflammation (also known as CLIP, or chronic low-grade inflammatory profile), oxidative stress, and impaired extracellular matrix remodeling are proposed to be common denominators for mechanisms underlying impairments to muscle health and decreased satellite cell functionality in diabetes mellitus.

Following a period of uncontrolled diabetes mellitus in humans (i.e., pre-diagnosis), insulin administration is the therapeutic standard, and is well studied in terms of regulating muscle protein turnover (Pain et al., 1983; Price et al., 1996; Charlton and Nair, 1998; Lee et al., 2004). What remains less known is the effect of insulin therapy on SCs. Insulin has been found to stimulate both proliferation and differentiation of SCs, with such evidence derived from a handful of *in vitro* studies (Ewton and Florini, 1981; Vandenburgh et al., 1991; Cassar-Malek et al., 1999). The paucity of data available from human diabetic muscle exposed to insulin treatment merits further consideration. In the absence of insulin, or poorly managed diabetic states, there may be a myriad of factors and processes stemming from the diabetic environment that have the potential to influence SC activity. After a review of many of these mechanisms, a select few are evident in both T1DM and T2DM. The precise modifications to skeletal muscle following diabetes onset is depicted along with the predicted mechanisms of action (Figure 1). These include, but are not limited to: oxidative stress, chronic low-grade inflammation, and impaired ECM remodeling. Though the impact of metabolic diseases on the changing metabolic needs of the muscle satellite cells as they move from quiescence through to differentiation is certainly of note, it is beyond the scope of this mini-review. We refer the readers to some excellent recent reviews (Fulco et al., 2008; Ryall, 2013) on this topic.

### Oxidative stress

Oxidative stress is evident in both T1DM (Aragno et al., 2004) and T2DM (Henriksen et al., 2010), and has been directly associated with elevated glucose concentrations (Bonnefont-Rousselot, 2002). Dysregulation of nitric oxide (NO) production also occurs, as hyperglycemia promotes the formation of reactive nitrogen species (RNS) to further exacerbate levels of oxidative stress (Zou et al., 2002). A shift in pro-oxidant/antioxidant balance is regarded in the pathogenesis of diabetes and its complications (Evans et al., 2002). Although an emphasis of research relating oxidative stress to skeletal muscle health has been on its modulation of protein turnover (Li et al., 1998; Zhou et al., 2001; Aragno et al., 2004), it is speculated that the concomitant increase in ROS and decrease in NO hinders satellite cell function. *In vitro* work has found that acute treatment of human muscle SCs with the ROS-inducing agent hydrogen peroxide (H_2_O_2_) led to reduced cell viability, shortened lifespan, and decreased proliferative capacity (Renault et al., 2002). In support of oxidative stress impairing myogenesis, Aragno et al. (2004) found that in response to muscle damage, the expression of critical myogenic factors (MyoD, myogenin, and Jun D) was reduced in STZ-diabetic rodents compared to non-diabetic rodents. Muscle creatine kinase and myosin expression were also impaired, suggesting that defects in the early phases of regeneration (i.e., satellite cell functionality) led to a cascade of events to further hinder muscle repair. It is interesting to note that oxidative stress has been implicated in the adipogenic conversion of muscle SCs (Vettor et al., 2009). Now whether this occurs within diabetic muscle has yet to be defined, however, given that the demonstrated impairments in myogenesis with diabetes appear to be linked to oxidative stress, it is clear that this area requires further investigation.

### Chronic low-grade inflammatory profile (CLIP)

With diabetes progression, a multitude of pro-inflammatory factors are elevated, constituting a state of chronic low-grade inflammation, or a chronic low-grade inflammatory profile (CLIP). The presence of this condition is evident in all forms of diabetes mellitus (Llauradó et al., 2012; Osborn and Olefsky, 2012) and is believed to occur as a result of the enhanced formation of advanced glycation end-products (AGEs; Tan et al., 2004; Ramasamy et al., 2005; Yan et al., 2008). The factors associated with CLIP can collectively and/or independently influence SC activity. While examination of each of these factors on SC function is beyond the breadth of this review, it is important to highlight a select few.

Chronic elevations of circulating Interleukin-6 (IL-6) are observed in T1DM and T2DM (Pradhan et al., 2001; Reis et al., 2012). While transient increases in IL-6 are associated with SC proliferation (Toth et al., 2011), chronically elevated IL-6 is correlated with significant decrements in muscle health (e.g., cancer cachexia; Roubenoff, 1997; McKay et al., 2013). Given the chronic elevations in IL-6 with diabetes, it is reasonable to surmise that impairments to SC functionality are occurring. Consistent with this hypothesis, obese diabetic individuals displayed significant impairments in IL-6 signaling within their skeletal muscle that persisted within the satellite cells even upon removal from the diabetic environment (Nielsen et al., 2012).

Akin to IL-6, tumor necrosis factor-α (TNF-α) functions as a key mediator of the inflammatory process. Not only is TNF-α correlated with diabetes progression (Csizuadia et al., 2012; Swaroop et al., 2012), it has also been found to alter insulin-mediated glucose uptake in muscle cells *in vitro* (Yoon et al., 2011), and has been implicated in the development of insulin resistance through studies knocking out its respective receptors (Uysal et al., 1997; Romanatto et al., 2009). With respect to SCs, TNF-α is thought to exert its effects through stimulation of factors that promote entry into the cell cycle (Li et al., 2003). In support of this, TNF-α treated myoblasts displayed an increased proliferative capacity while differentiation was hindered (Alter et al., 2008).

The presence of CLIP, as found in diabetes, will undoubtedly alter skeletal muscle homeostasis. This emerging and exciting new area of interest, though still in its infancy, presents an intriguing avenue for further therapeutic investigations.

### Impaired extracellular matrix (ECM) remodeling

Studies have found that central constituents of the plasminogen system are required for normal growth and repair within a variety of tissues types, including skeletal muscle (Romer et al., 1996; Lluís et al., 2001; Shimizu et al., 2001). Within muscle, inhibition of PAI-1 (a critical inhibitor of the plasminogen system) was found to increase MyoD expression and accelerate muscle repair (Koh et al., 2005). Of particular note, elevated ECM levels have been demonstrated in a variety of diabetic tissues (Berria et al., 2006; Krause et al., 2011). Excessive ECM levels are likely the result of altered protein expression (Lecker et al., 2004), especially in regards to matrix metalloproteinases (Hopps and Caimi, 2012). The improper turnover of ECM proteins may also hinder growth factor signaling, further impeding myogenesis (Gopinath and Rando, 2008). While the aforementioned studies identify that aspects of muscle health are clearly subject to modification with diabetes mellitus, one must also account for diabetic-induced changes to the environment in which the muscle SCs reside. The adverse remodeling of the ECM in diabetic muscle, as evidenced by increased collagen presence, will inevitably affect SC functionality and its capacity to migrate within regenerating muscle (Krause et al., 2013). These defects are also prevalent in senescent skeletal muscle (Franceschi, 2007; Kurtz and Oh, 2012; Vasilaki and Jackson, 2013). Thus, potential therapies to attenuate negative alterations to SC behavior with diabetes onset may also function to mitigate sarcopenia.

## Significance and conclusions

Diabetes mellitus is a global health concern. While diabetes mellitus begins as a result of an impairment in insulin signaling (deficiency/resistance), numerous other factors quickly become altered making the pathogenesis of diabetic complications multi-faceted. Here we provide an overview of the importance of the muscle SC, the impact of T1DM and T2DM on this cell population, and potential “common” mechanisms for altered SC function. Based on the limited number of studies to date, it is evident that various stages of the myogenic process are affected by diabetes mellitus and impairments to SC function are occurring. Given the vital role of these cells in the lifelong maintenance of skeletal muscle, and the importance of a physically and metabolically healthy skeletal muscle mass in attenuating the morbidity and mortality associated with diabetes mellitus, a comprehensive understanding of SC in the diabetic environment is of fundamental significance. Identifying the proponents that attenuate normal SC function in diabetes mellitus will lead to the development of therapies that restore SC activity in order to sustain muscle health, and subsequently attenuate other diabetic complications.

### Conflict of interest statement

The authors declare that the research was conducted in the absence of any commercial or financial relationships that could be construed as a potential conflict of interest.
